# Nutrition Quality Parameters of Almonds as Affected by Deficit Irrigation Strategies

**DOI:** 10.3390/molecules24142646

**Published:** 2019-07-21

**Authors:** Leontina Lipan, Alfonso Moriana, David B. López Lluch, Marina Cano-Lamadrid, Esther Sendra, Francisca Hernández, Laura Vázquez-Araújo, Mireia Corell, Ángel A. Carbonell-Barrachina

**Affiliations:** 1 Universidad Miguel Hernández de Elche, Escuela Politécnica Superior de Orihuela, Department of Agro-Food Technology, Research Group “Food Quality and Safety”, Carretera de Beniel, km 3.2, 03312-Orihuela, Alicante, Spain; 2 Departamento de Ciencias Agroforestales, ETSIA, Universidad de Sevilla, Carretera de Utrera, km 1, 41013 Sevilla, Spain; 3 Unidad Asociada al CSIC de Uso Sostenible del Suelo y el Agua en la Agricultura (US-IRNAS), Crta de Utrera km 1, 41013 Sevilla, Spain; 4 Universidad Miguel Hernández de Elche, Escuela Politécnica Superior de Orihuela, Department of Agro-Environmental Economics, Carretera de Beniel, km 3.2, 03312-Orihuela, Alicante, Spain; 5 Universidad Miguel Hernández de Elche, Escuela Politécnica Superior de Orihuela, Department of Plant Science and Microbiology, Research Group “Plant Production and Technology”, Carretera de Beniel, km 3.2, 03312-Orihuela, Alicante, Spain; 6 BCCInnovation, Technological Center in Gastronomy, Juan Avelino Barriola 101, 20009-Donostia-San Sebastián, Gipuzkoa, Spain; 7 Basque Culinary Center, Mondragon Unibersitatea, Juan Avelino Barriola 101, 20009-Donostia-San Sebastián, Gipuzkoa, Spain

**Keywords:** sugars, antioxidants, phenols, volatile compounds, hydroSOStainable products, *Prunus dulcis*

## Abstract

The influence of full irrigation, double-regulated (RDI) and sustained deficit irrigation (SDI) treatments on almond quality was assessed by analyzing different parameters: sugars, organic acids, antioxidant activity, total phenolic content (TPC), and volatile compounds. Almond quality studies for plants submitted to water stress are scarce, and it is essential to understand the biochemical responses of plants to water stress in maintaining fruit yield and quality. Citric acid, sucrose, antioxidant activity, and TPC were not affected by the application of studied deficit irrigation strategies (DI). An increase in malic acid and a decrease in glucose was observed for stressed samples (T3 and T4), while a higher number of total volatiles compounds was found for moderate RDI (T2). Using deficit irrigation strategies, the almond yield and quality was not changed, and in fact, some parameters, such as glucose and key volatile compounds, slightly increased under moderate RDI. This finding might encourage farmers to implement these strategies and contribute to sustainable agriculture.

## 1. Introduction

Fresh (non-salty and adequate for irrigation) water is a limited resource and the uncertainty of the remaining amount for the next generation has it this a dramatic global risk factor [1,2]. Agriculture is the primary use of fresh water worldwide because more than 40% of food production comes from irrigated fields, and it is in a weak position due to its susceptibility to weather (temperatures and precipitation) changes [3]. Mediterranean agriculture is a perfect model of arid and semiarid farming, in which fields must deal with limited irrigation water supplies due to scarce rain leading to periodic drought and competitiveness with other productive sectors, such as tourism [4,5]. For this reason, identifying agricultural practices that increase water use efficiency is necessary to be able to develop a sustainable agricultural system [6]. 

When considering the cultivated surface area, almond (*Prunus dulcis*) is the third most cultivated tree in Spain, and the major tree nut crop in the Mediterranean area [4,6]. Although it is a drought resistant species, it is believed that irrigation is needed to improve yield and fruit quality [7]. However, many authors showed that irrigation water could be reduced, maintaining or even slightly improving fruit quality by using deficit irrigation (DI) strategies [6,8,9,10,11,12,13,14]. Oher nuts, such as pistachio, are also considered a great alternative for arid and semiarid areas, helping farmers to increase income by saving irrigation water but having minimum impact on yield and fruit quality when the adequate irrigation strategy is applied [10,15]. A DI strategy refers to an agricultural practice in which irrigation water is applied below the crop evapotranspiration (ET; the soil evaporation and plant transpiration losses) needs. Regulated deficit irrigation (RDI) is a DI strategy in which the amount of irrigation water is reduced during a specific period when the crop is less stress sensitive. On the other hand, in sustained deficit irrigation (SDI), a uniform and reduced amount of water is applied during the whole growing cycle of the plant or tree, developing a progressive stress in the plant [6]. Products obtained from plants submitted to a controlled water stress are called hydroSOStainable (*hydroSOS*) [2,13,14,15,16,17,18,19].

Sugars and organic acids of “Vairo” almonds under water stress conditions are scarce in the scientific literature, and although organic acids play a limited role in the quality of almonds, sugars are essential for good flavor and taste [20]. The antioxidant activity (AA) is related to the ability of almonds to reduce pro-oxidant agents; AA is considered a key quality feature by consumers because an increase in the consumption of antioxidant compounds was associated with reduced obesity in women and also helped in reducing the risk of stroke and cardiovascular diseases, and even some cancer types [21]. Almond polyphenols are mostly found in its skin, with values ranging from 9.10 to 32.1 g kg^−1^ (skin values obtained from blanched and roasted almonds, freeze-dried or dried in a hot-air oven at 60 °C) [22]. However, almond skin is often removed by blanching for commercial reasons and this unit operation will drastically reduce the polyphenol content. Volatile compounds are responsible for the characteristic flavor properties of raw and processed almonds and contribute to their high consumer acceptance. Benzaldehyde is one of the main volatiles in bitter almond, but in general, it is found in very low amounts in sweet almonds [23].

The aim of this work was to evaluate the influence of RDI and SDI conditions on quality parameters of almonds, namely on the content of sugars, organic acids, total phenolic and volatile compounds, and on the antioxidant activity. Optimizing the water resources in almond farming in the Mediterranean area of Spain is essential for the sustainability of Spanish agriculture.

## 2. Results and Discussion

### 2.1. Irrigation

**Figure 1:**
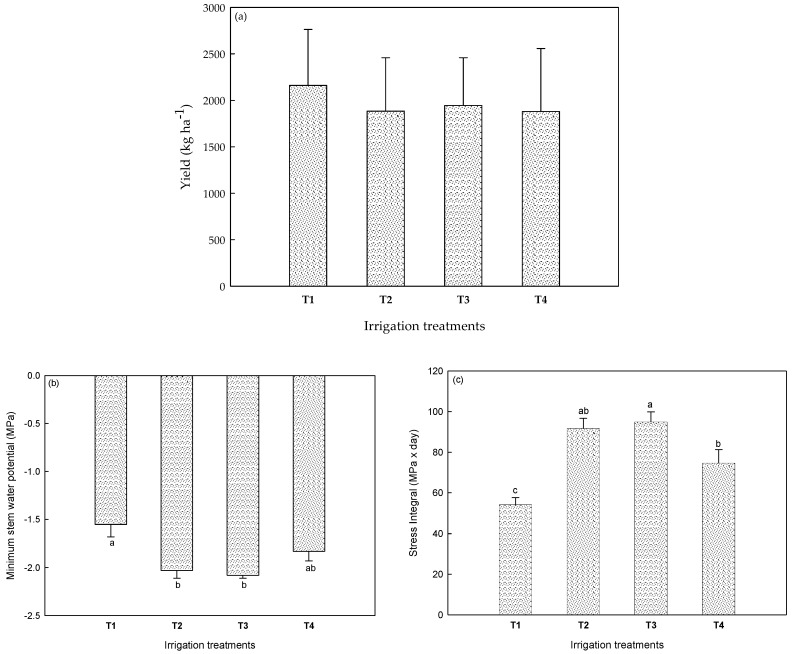
Almond yield (**a**), minimum stem water potential (**b**), and water stress integral (**c**) for each treatment. Bars with the same letter were not significantly different ((**a**) *p* < 0.001; (**b**) *p* < 0.01; (**c**) *p* < 0.001), according to Tukey’s least significant difference test.

Four irrigation treatments, including a control one, were applied at different stress levels. The amount of irrigation water applied within this study ranged from 100 to 433 mm (T1 = 433 ± 26; T2 = 148 ± 24, T3 = 103 ± 3.0, and T4 = 114 ± 13 mm). Figure 1 shows the almond yield for each treatment (Figure 1a), the minimum values of the stem water potential (Figure 1b), and the waters stress integral (Figure 1c) for each treatment. It is very important to highlight that almond yield was not significantly (*p* < 0.05) affected by any of the treatments under study. These results agreed with previous studies on almonds and pistachios grown under regulated deficit irrigation [10,24]. Regarding almonds, the study was done for the “Marta” genotype over five years, and although a yield decrease was observed for the first and fourth years, no differences were seen for second, third, and fifth years [24]. Moreover, authors working with pistachio from Kerman cultivar concluded that the nut yield was not affected by RDI in any of the two seasons under the studied conditions [10]. The implementation of deficit irrigation DI strategies was possible by controlling the crop water status through midday stem water potential. As observed in Figure 1b,c, T1 showed the lowest levels of stress with a value of −1.55 MPa for the minimum stem water potential (SWP) and a stress integral (SI) of 54.2 MPa × day, while T3 (severe RDI) displayed the highest levels of stress for both parameters (MinSWP = −2.08 MPa; SI = 94.9 MPa × day).

### 2.2. Sugars and Organic Acids

Sucrose was the main soluble sugar found in almonds, representing 85–91% of the total carbohydrates, due to its accumulation during ripening and because many other reducing sugars are substrates for its synthesis [25]. Table 1 shows that the results of the sucrose content ranged from 33 to 35 g kg^−1^, being significantly similar among treatments. Previous studies reported a sucrose increase with irrigation (total water supply of ~10 m^3^ tree^−1^ during summer) in almonds from “Ferragnes”, “Texas”, and “Guara” genotypes, but different irrigation strategies were applied [20,25]. On the contrary, other authors observed a higher sucrose content in non-irrigated almonds from “Marta” variety and linked it to the plant adaptive physiological responses to water stress, while a significant similarity between those irrigated at full (110%) ET, 50% ET periodically supplied to only one side of the root system for the entire year (PRD), and RDI (irrigation at 100% of ET for the entire year and at 30% of ET from early June until harvest) was observed [26].

Glucose, fructose, sorbitol, raffinose, and inositol were other monosaccharides found previously in almonds [25], however in this study, glucose and fructose were the only reducing sugars identified. While fructose was found only in trace amounts (data not shown), glucose content presented statistically significant higher values for T1 and T2 (moderate RDI) treatments. Sánchez Bel et al. [25] in studies about almonds (“Guara” genotype), and Nahar et al. [27] in tomatoes, concluded that water stress enhanced the sweetness of tomatoes by increasing their glucose content. These results might be related to the osmotic adjustment, which can be activated by accumulation of solutes (rich in hydroxyl (-OH) groups, such as sugars, proline, etc.) in the cytoplasm under stress conditions. This biochemical mechanism aids plants in naturalizing to dry and saline conditions by protecting the cellular membrane, protein, and enzymes against dehydration [28]. Consequently, the osmotic adjustment enhances the capacity to maintain positive turgor, increasing the sugars and organic acid [27]. Egea et al. [6] and Cornacchia et al. [26] obtained no statistically significant differences among fully irrigated and DI strategies in almond trees.

The main identified organic acids (Table 1) were citric and malic. Citric acid was not affected by DI, as other authors have previously reported [6]. On the contrary, 0.4% increase of citric acid content was observed in drip–irrigated “Guara” almonds (16.8 m^3^ year^−1^) with respect to non-irrigated almonds [25]. An increase of 2.4 and 4.2 g kg^−1^ of malic acid content for T3 and T4 (SDI), respectively, was observed; this experimental finding agreed with previous studies on tomatoes and grapes, in which water stress enhanced their quality by raising the concentration of important organic acids [27]. However, a decrease of malic acid content in “Guara” almonds under non-irrigated conditions [11] and in “Marta” almonds under partial root zone (PRD) conditions (irrigation supplied at 50% ET during the whole growing season) was observed [25]. Malic acid was stable for moderate RDI and similar results were reported in “Marta” almonds under RDI (trees were irrigated at 50% ET during kernel filling and at 100% ET for the rest growth period) [6]. Studies in organic acid content in almonds under water stress conditions are scarce, although it seems that organic acids may play a limited role in the quality of almonds.

### 2.3. Antioxidant Activity (ABTS^+^, DPPH^•^, and FRAP methods) and Total Phenolic Content

Table 2 shows the results of antioxidant activity and total phenolic content (TPC) analyzed in raw kernel, blanched kernels, and almond skin. An enormous difference in antioxidant activity and polyphenols for raw almonds, blanched, and skin was observed, and mean values of all treatments were: (i) ABTS^+^ (2.2-azino-bis) = 9.5, 1.5, and 36 mmol Trolox kg^−1^, respectively; (ii) DPPH^●^ (2,2-diphenil-1-picrylhydrazyl) = 28, 20, and 33 mmol Trolox kg^−1^, respectively; (iii) FRAP (ferric reducing ability of plasma) = 3.9, 0.5, and 62 mmol Trolox kg^−1^, respectively.

The TPC found in this study for raw kernel (5.5 g gallic acid equivalents (GAE) kg^−1^, mean value of all treatments) was higher than those previously reported by other authors (range from 0.6 to 1.9 g GAE kg^−1^) but lower than that reported by Lin et al. [29] (7.5 g GAE kg^−1^) for the almonds from Almond Board of California. The difference could be attributed to factors such as variety, geographical area, or agricultural practices [21,30]. Blanched kernel TPC (0.5 g GAE kg^−1^) was similar to that reported in literature (0.7 g GAE kg^−1^) for almonds in general [30]. Finally, a mean TPC value of 13 g GAE kg^−1^ was found on almond skin and similar values were reported for American almonds (11–17 g GAE kg^−1^), but lower values were found for Spanish almonds (26 g GAE kg^−1^) [22].

The total dietary intake of polyphenols is estimated to be around 1 g [31]. The results of the present study showed that, for example, ~30 g of almonds (~20 kernels of “Vairo” genotype cultivated in Sevilla, Spain) provides ~170 mg of total phenols and represents almost 20% of the total dietary polyphenols intake.

Antioxidant activity and TPC were not significantly affected by the irrigation treatment in the raw almond. Regarding the blanched almonds, T4 was found to have the lowest antioxidant activity measured with ABTS and the lowest TPC. Blanched samples from the moderate RDI (T2) showed the highest TPC and the antioxidant activity was similar to the control (for the ABTS and DPPH methods). Regarding the almond skin, the TPC and antioxidant activity (ABTS and DPPH) were not affected by the irrigation treatments, except for FRAP method, which showed that T2 was similar to the control and T4 had the lowest antioxidant activity. These results agreed with Cano Lamadrid et al. [32] and Sánchez-Rodríguez et al. [12] in studies investigating effects of deficit irrigation on quality and functional profiles of table olives (hydroSOStainable olives) and pomegranates, respectively [9,12,33]. An increase, however, in polyphenols and antioxidant activity was observed for lettuce growing under water stress conditions [34] and grapes from clusters exposed to RDI (compared with SDI), which might happen due to the reduction of canopy leaf area of vines under RDI conditions [35].

### 2.4. Volatile Compounds

Twenty-six compounds were identified and quantified in the volatile profile of “Vairo” almonds. A total of 10 alcohols, 9 alkanes, 3 aldehydes, 1 terpene, 1 ketone, and 1 organic acid were identified and are presented in Table 3, together with their retention time, retention indices, and their odor descriptors, while Table 4 presents the contents of the volatile compounds. Alcohols, which were the most abundant volatiles (0.82 mg kg^−1^), are released by enzymatic reactions in raw almond and contribute to the characteristic sweet aroma and to the consumer acceptance [23]. Most of the alcohols were not affected by the studied treatments, except 1-hexanol, which had the highest content, 0.40 mg kg^−1^, in T2 almonds. High levels of hexanol content was also found by other authors in Nonpareil almonds extracted with a similar method [23]. It is known that hexanol increases with the almond ripening and is associated with herbal odor (fruity, alcoholic, sweet, green notes) and green flavor (fruity, apple skin, oily) [11,36]. Garcia Esparza et al. [11] in studies about DI in grapes concluded that watering during post-veraison (the change of grapes color) at 75% of the crop ET (irrigation was applied to replace 75% of crop ET) compared to rain fed decreased the alcohol content (from 4.08 to 3.91 mg g^−1^) and increased that of the aldehydes (hexanal from 0.43 to 0.50 mg g^−1^), which produced herbaceous (non-desirable) aromas in wines [11].

From the alkanes (2.2 mg kg^−1^ mean values), statistically significant differences among studied treatments were found for pentamethyl heptane and tridecane. Pentamethyl heptane increased in T3 and T4 samples and tridecane in T2 and T3 samples. Previous studies showed that tridecane was formed by decarboxylation of myristic fatty acid (C14:0) [39] and this fact could be explained by the higher content of tridecane in T2 and T3, which can be induced by the water stress produced in the almond trees by RDI and could affect the plant metabolism.

Limonene was the only terpene found, and statistically significant differences were observed among the samples. Moderate RDI (T2) increased the limonene content, which is associated with fresh, citrus, and sweet notes. This result agreed with Carbonell et al. [10], who observed a higher content of limonene (from 12.4% to 14.8%) in pistachio under moderate RDI [10].

Benzaldehyde, which is the major volatile compound in bitter almond, was not predominant in these almonds, and this might be attributed to the low content of amygdalin [23]. Amygdalin is a cyanogenic glycoside naturally produced in almond, and it is the benzaldehyde precursor [23]. No statistically significant differences between the control and DI treatments were observed, implying that the sensory quality might not be affected by DI strategies. The finding agreed with other authors in studies of pistachio under RDI [10].

The total content of volatile compounds was significantly different among samples. Almonds from moderate RDI showed a higher total content (4.39 mg kg^−1^) as compared to the control samples (2.50 mg kg^−1^). Similar results (4.36 mg kg^−1^) were observed for raw almonds (mixture of “Butte” and “Padre” varieties) [40]. The aroma of raw almonds is, in general, weak, and a low total content of volatile compounds is usually expected. However, this content can be increased during roasting (6.17 mg kg^−1^ after 28 min of roasting; 11.4 mg kg^−1^ after 33 min of roasting, and 16.0 mg kg^−1^ after 38 min of roasting) [40] due to the Maillard and lipid oxidation reactions [40,41,42].

In this study, a reduced number and content of aldehydes was found, confirming the freshness of the studied almonds. In previous studies, aldehydes (e.g., hexanal) levels significantly increased, from 0.42 mg kg^−1^ in raw Butte and Padre almonds to 0.98 mg kg^−1^ in almonds roasted for 28 min at 138 °C and to 1.63 mg kg^−1^ after 24 weeks of storage at 35 °C [43]. During storage, and due to lipid oxidation reactions [44], hexanal increased, and this is why this compound (hexanal) is considered an indicator of oxidation or rancidity (low degree of freshness) in nuts and nut oils. The freshness (low level of rancidity) of all four almond samples was confirmed by the low content of hexanal and nonanal found [44,45]. Both hexanal and nonanal values showed no statistical significant differences among treatments, as well as a low content of hexanal and a similar content of nonanal when compared to the literature [46].

### 2.5. Pearson’s Correlation Coefficients

Table 5 shows the Pearson’s correlation coefficients among studied variables with significant differences among treatments. A positive and significant correlation was observed between stress integral (SI) and (i) TPC (R = 0.72; *p* < 0.01), (ii) 2-butanol (R = 0.57; *p* < 0.05), and (iii) total volatile content (R = 0.75; *p* < 0.001). A positive correlation between the TPC and water stress levels in leaves of *Solanum villosum* and roots of *Solanum scabrum* has been reported [47]; this same positive correlation was also observed in tomatoes plants and maize [48]. Water stress can create damage in plants due to formation of reactive oxygen species (ROS) and to the alteration of the water–plant relationship [48]. The degree to which the plant can avoid or soften the physiological processes determines the resistance degree to water stress of each plant species [48]. Volatile compounds, which confers the typical almond aroma, were also found to be positively correlated with water deficit in different plants, such as grapevines, apples, tomatoes, and strawberry [49]. The increase in the total volatile compound value might be associated with the metabolic responses that deal with (i) high levels of light energy and (ii) formation of oxidative compounds under drought [49].

A negative correlation was found between the contents of glucose and malic acid (R = −0.89; *p* > 0.05). Partially similar results were reported in other studies on plums, apricots, and apples [50,51]. This negative correlation is normally observed during the ripening process, because glucose and fructose increased due to the reaction of invertase enzyme through glycolysis, while the organic acids decreased because they are used in respiration and are converted into sugars [52].

A significant correlation was observed between the TPC and the total content of volatile compounds (R = 0.76; *p* < 0.001); this same trend was also found in olives [53].

A statistically significant correlation was found between the contents of linoleic acid and hexanal (R = 0.83; *p* < 0.05), and between oleic acid and nonanal (R = 0.68; *p* < 0.05). This agreed with previous studies, in which positive correlations were reported for the content of linoleic acid and the production of aldehydes ((*E*)-2-heptenal, (*E*)-2-octenal, (*E*,*E*)-2,4-decadienal and (*E*,*E*)-2,4-nonadienal) [44]. The oils containing linoleic (C18:2) and linolenic (C18:3) acids are converted into hexanal, (*E*)-2-hexenal, or (*Z*)-3-hexenal through the enzymatic pathway of lipoxygenase and hydroperoxide lyase [46]. Thus, hexanal is derived from 13-hydroperoxide, which is one of the most abundant hydroperoxides produced by autooxidation of linoleic acid, and it is directly linked to the development of oxidative off-flavors [44]. On the other hand, nonanal is produced from the oleic acid (C18:1) [44,45].

## 3. Materials and Methods

### 3.1. Plant Material, Growing Conditions and Experimental Design

The experiment was performed during the 2017 season at the commercial farm “La Florida” (37.23° N, −5.91° W, Dos Hermanas, Seville, Spain). The almond (*Prunus dulcis*) orchard was 7 years-old at the beginning of the experiment. There were 2 almond cultivars in the orchard, “Guara” and “Vairo”, and the tree spacing for both cultivars was 6 m × 8 m. The experimental plots had 4 lines of 3 trees and measurements were performed in the central trees of the “Vairo”. The trees were irrigated with a line of drip emitters (3.8 L h^−1^) separated by 0.4 m. Irrigation scheduling was performed daily.

**Figure 2:**
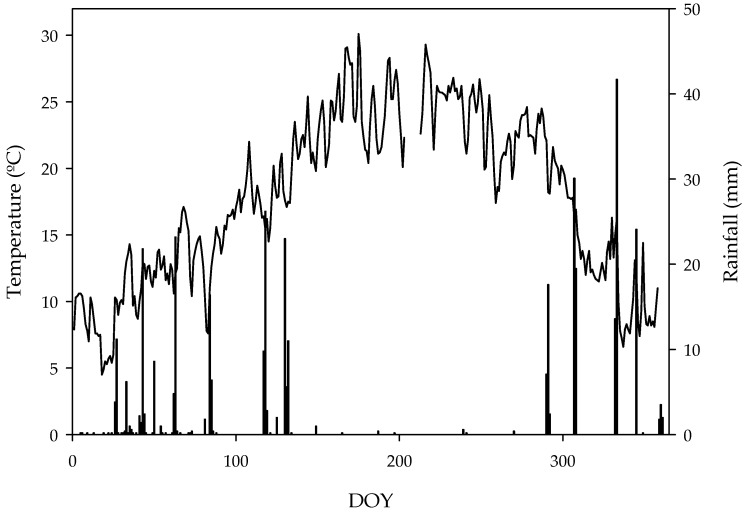
Annual pattern of daily mean air temperature and rainfall. Data were obtained from the “IFAPA Los Palacios” station, which is approximately 6 km away from the experiment site. This meteorological station is part of the Andalusian agroclimatic stations network (Junta de Andalucía).

The seasonal weather data were obtained from the “Instituto de Investigación y Formación Agraria (IFAPA) Los Palacios” station, in the Andalusian weather stations network (Figure 2). This station is about 6 km away from the experimental orchard. The data for 2017 was typical of Mediterranean zones, with null rainfall during the summer period and warm winters.

The irrigation was scheduled according to measurements performed using a pressure chamber (PMS Instrument Company, Albany, OR, USA) and the threshold values of midday stem water potential (SWP) were measured to evaluate the level of plant stress. Three irrigations treatments were established together with a control treatment:

- Full irrigation (T1), to assure the estimated ET during the entire growing season.
- Moderate RDI (T2); in the period of kernel filling, almonds were irrigated when SWP < −1.5 MPa, and for the rest of the time, trees were irrigated to keep a SWP as the baseline proposed by McCutchan and A Shackel [54].
- Severe RDI (T3); the same as T2, except trees were irrigated when SWP < −2 MPa during kernel filling.
- SDI (T4); a lower amount of water was distributed uniformly throughout the year.

Equation (1) was used to calculate the stress integral (SI) and min ψ_stem_ represented the average of minimum SWP for any interval, while *n* was the number of days interval:

(1) $$\mathrm{SI}=|\sum \left({\min\;\psi }_{\mathrm{stem}}-\left(-0.2\right)\right)\;\times \;n$$

The harvest was done in August using a self-propelled trunk shaker with collector. Each treatment was harvested separately, and almonds were sun-dried to reach a moisture content below 5%, and then delivered to Miguel Hernández University for quality analyses. Almonds were shelled, and the kernels were ground, vacuum packed, and frozen until analysis.

### 3.2. Sugars and Organic Acids

High-performance liquid chromatography (HPLC) equipment was used to identify and quantify the sugars and organic acids as previously described by Lipan et al. [2] with some modification. Almond finely ground (1 g) in a Moulinex grinder (AR110830) for 10 s was homogenized with 5 mL of phosphate buffer 50 mM (pH = 7.8) with an homogenizer (Ultra Turrax T18 Basic) during 2 min at 11,300 rpm, while the tube was maintained in an ice bath, then was centrifuged (Sigma 3–18 K; Sigma Laborzentrifugen, Osterode and Harz, Germany) for 20 min at 15,000 rpm and 4 °C and was filtered (0.45 μm Millipore membrane filter). The filtered supernatant (10 μL) was injected into a Hewlett Packard (Wilmington DE) series 1100 (HPLC) using 0.1% ortophosphoric acid elution buffer. Sugars were measured using a Supelcogel TM C-610H column (30 cm × 7.8 mm) with a pre-column (Supelguard 5 cm × 4.6 mm; Supelco, Bellefonte, PA) and the detection was carried out with a refractive index detector (RID). Organic acids were separated in the same HPLC condition as sugars and absorbance was measured at 210 nm with a diode-array detector (DAD). Calibration curves were run in triplicate injection using standards of different organic acids and sugars provided by Sigma (Poole, UK). Analyses were run in triplicate and results were expressed as g kg^−1^ dw.

### 3.3. Fatty acids Analysis

Fatty acids methyl esters (FAMEs) were prepared as described by Lipan et al. [2] with some modification, while identification and quantification were done according to Tuberoso et al. [55]. Briefly, 40 mg of ground almond were saponified with 100 µL of dichloromethane (Cl_2_CH_2_) and 1 mL of sodium methoxide solution and kept for 10 min at 90 °C. Boron trifluoride (BF_3_) methanolic (1 mL) was added, followed by 30 min of reaction in darkness. FAME extraction from the mixture was done with 1.5 mL hexane. The separation of FAMEs was conducted using a Shimadzu GC17A gas chromatography coupled with a flame ionization detector and a DB-23 capillary column (30 m length, 0.25 mm internal diameter, 0.25 µm film thickness) J&W Scientific, Agilent Technologies. Helium gas was used as the carrier with a flow rate of 1.1 mL min^−1^, and 35 mL min^−1^ at the make-up point. The temperatures of the injector and detector were 240 and 260 °C, respectively. The injection volume was 0.8 µL (split ratio 1:20). Finally, the temperature program was: initial temperature 100 °C held for 1 min, temperature gradient of 3 °C min^−1^ until 220 °C, followed by a gradient of 5 °C min^−1^ until 245 °C, and kept at 245 °C for 1 min. FAME peaks identification was performed by comparing the retention times of the FAME Supelco MIX-37 standards and the results were expressed quantitatively as g kg^−1^ concentration using methyl nonadecanoate as internal standard.

### 3.4. Antioxidant Activity and Total Phenolic Content

The antioxidant activity and total phenolic content was carried out not only for raw kernel but also for blanched almond and its skin. The blanching process consisted of almond immersion in boiling water (100 °C) for 2 min, followed by manual skin removal. The method of extraction consisted of 0.5 g of sample being sonicated with 10 mL of extractant (MeOH/water (80:20, *v*/*v*) + 1% HCl at 20 °C) for 15 min and stored for 24 h at 4 °C. The next day, the mixture was sonicated again under the above-mentioned conditions, then, it was centrifuged at 10,000 rpm for 10 min.

The antioxidant activity of the obtained extract was measured using 3 methods: DPPH^●^, ABTS^+^, and FRAP. DPPH^●^ (2,2-diphenyl-1-picrylhydrazyl) free radical was used to determine the radical scavenging activity of the sample as described by Brand-Williams et al. [56]. A brief description of the process is that 10 μL of the sample were mixed with 40 μL of MeOH and 990 μL of the free radical solution, shaken, and placed in darkness for 15 min. Later, the sample absorbance was measured at 515 nm. Moreover, 10 μL of the sample supernatant was mixed with 990 μL solution of ABTS^+^ (2,2-azinobis-(3-ethylbenzothiazoline-6-sulfonic acid)) or FRAP (ferric reducing antioxidant power) free radicals to determine the free radical scavenging capacity of the sample. After 10 min of reaction, the sample absorbance was read at 734 nm for ABTS^+^ method and 593 nm for FRAP. All measurements were carried out in an ultraviolet-visible (UV-vis) spectrophotometer (Helios Gamma model, UVG 1002E; Helios, Cambridge, UK). The quantification was done according to the calibration curve of Trolox, prepared in a concentration ranging from 0.5 to 5.0 mmol Trolox L^−1^. The linearity was above *R^2^* = 0.998 and results were expressed in mmol Trolox kg^−1^ [9,56].

Total polyphenolic content (TPC) was determined using the Folin-Ciocâlteu colorimetric method, in which 100 μL of sample supernatant were mixed with 200 μL Folin-Ciocâlteu reagent and 2 mL of H_2_O_2_. This mixture was stored at 22 °C for 3 min and 1 mL of 20% sodium carbonate was added, followed by incubation for 1 h at room temperature. Later, the mixture absorbance was measured at 765 nm in the above-mentioned equipment. The results were calculated according to the gallic acid calibration curve and were expressed as gallic acid equivalents (GAE), g GAE kg^−1^ [9,56].

### 3.5. Volatile Compounds Analysis

Volatile compounds were extracted using headspace solid phase microextraction (HS-SPME). For the extraction, 2 g of grinded almond and 50 μL of β-ionone (100 mg L^−1^) were placed in a hermetic vial with polypropylene cap and PTFE(polytetrafluoroethylene)/silicone septa and was used as an internal standard; this internal standard was used for the semi-quantification of the volatile compounds, as no calibration curve was done for each of the compounds reported in this study. The vial was placed in a water bath at controlled temperature to assure the vial was 40 °C, which was needed to simulate the mouth temperature when chewing almonds. Once the temperature was reached and was constant, a 50/30 μm Divinylbenzene/Carboxen/Polydimethylsiloxane (DVB/CAR/PDMS) fiber was introduced in the headspace of the vial for 50 min. This fiber is characterized by high capacity of trapping volatile compounds from fruits and nuts. Moreover, the fiber was desorbed for 3 min in the injector port of a gas chromatograph Shimadzu GC-17A (Shimadzu Corporation, Kyoto, Japan) coupled with mass spectrometer (MS) detector Shimadzu GC-MS QP-5050A used for the volatile compounds identification. The GC-MS was equipped with a SLB-5ms Fused Silica Capillary Column of 30 m × 0.25 mm × 0.25 µm film thickness, 5% diphenyl, and 95% dimethyl siloxane (Supelco Analytical). For the analyses, helium was used as gas carrier at a flow rate of 0.7 mL min^−1^ in splitless mode. The oven program was: (a) initial temperature 40 °C, (b) rate of 2.0 °C min^−1^ to 145 °C, (c) rate of 25 °C min^−1^ from 145 to 300 °C and hold for 90 s. In addition, the injector was kept at 230 °C, while the detector at 320 °C. The volatile compounds identification was done by using 3 methods: (a) retention indices, (b) Gas Chromatography - Mass Spectrometry (GC-MS) retention times of authentic chemicals, and (c) mass spectra (authentic chemicals and NIST05 spectral library collection) [38].

## 4. Statistical Analysis

The statistical analyses were done by using one-way analysis of variance (ANOVA), and data were submitted to Tukey’s multiple range test to compare means. Statistically significant differences were considered when *p* < 0.05 and were studied using XLSTAT Premium 2016 (Addinsoft, New York, USA). Pearson’s correlation was carried out with the same program in which data were subjected to Correlation Tests. For figures preparation, Sigma Plot 11 was used.

## 5. Conclusions

Based on the literature search, this study was the first to evaluate the quality parameters (sugars, organic acids, antioxidant activity, and total contents of phenolic and volatile compounds) of hydroSOStainable “Vairo” almonds under regulated (RDI) and sustained (SDI) deficit irrigation. Almond yield was not affected either by RDI or SDI. Almonds are a very good source of polyphenols (5.5 g GAE kg^−1^; mean values for all treatments), and regarding the antioxidant activity and total phenolic compounds, there were no significant differences among irrigation treatments. Moderate regulated deficit irrigation led to almonds with high glucose content (potentially linked with almond sweetness) and total content of volatile compounds (potentially linked with almond odor, aroma, and flavor), implying a high sensory quality. Consequently, almond quality can be improved if the water stress in the plant is induced in a controlled way, contributing to sustainable agriculture with greater benefits for the farmers through commercialization of these almonds as hydroSOS products.

## Figures and Tables

**Table 1:** Sugars and organic acid profiles and contents (g kg^−1^ dry weight (dw)) of raw almonds as affected by deficit irrigation treatments.

Treatment	Sugars		Organic Acids	
	Sucrose	Glucose	Citric	Malic
(g kg^−1^ dw)				
**ANOVA Test** **^†^**				
	NS	***	NS	**
**Tukey’s Multiple Range Test** **^‡^**				
**T1**	33.31 ± 0.69	12.03 ± 0.53 a	2.27 ± 0.04	8.81 ± 0.47 b
**T2**	34.29 ± 0.49	11.78 ± 0.33 a	2.23 ± 0.04	9.02 ± 0.51 b
**T3**	35.47 ± 0.68	5.58 ± 0.28 b	2.31 ± 0.05	11.18 ± 0.96 ab
**T4**	35.12 ± 2.31	6.38 ± 0.53 b	2.36 ± 0.07	13.02 ± 1.33 a

Note: ^†^ NS = not significant at *p* < 0.05; * and ** significant at *p* < 0.05 and 0.01, respectively; ^‡^ Values (mean of 8 replications) followed by the same letter, within the same column, were not significantly different (*p* < 0.05), according to Tukey’s multiple range test.

**Table 2:** Antioxidant activity (mmol Trolox kg^−1^) and total phenolic content (mg gallic acid equivalents, GAE kg^−1^) of almond, as affected by deficit irrigation treatments.

	ABTS^+^ (mmol Trolox kg^−1^)	DPPH^●^ (mmol Trolox kg^−1^)	FRAP (mmol Trolox kg^−1^)	TPC (g GAE kg^−1^)
ANOVA *^†^*				
Raw almond	NS	NS	NS	NS
Blanched almond	*	NS	*	*
Skin	NS	NS	***	NS
Raw almonds ^‡^				
T1	9.24 ± 0.84	26.1 ± 1.10	5.00 ± 0.56	5.06 ± 0.22
T2	10.24 ± 0.66	29.3 ± 0.58	3.05 ± 0.47	5.83 ± 0.28
T3	9.04 ± 0.68	27.8 ± 0.10	3.22 ± 0.53	5.80 ± 0.34
T4	9.61 ± 0.33	27.4 ± 0.96	4.38 ± 0.32	5.39 ± 0.23
Blanched almonds ^‡^				
T1	1.65 ± 0.06 a	19.8 ± 0.52	0.50 ± 0.01 b	0.49 ± 0.06 b
T2	1.44 ± 0.07 ab	19.6 ± 1.00	0.53 ± 0.01 ab	0.71 ± 0.05 a
T3	1.71 ± 0.01 a	21.9 ± 0.56	0.60 ± 0.02 a	0.53 ± 0.01 b
T4	1.30 ± 0.12 b	19.0 ± 0.09	0.57 ± 0.03 ab	0.42 ± 0.06 c
Almond skin ^‡^				
T1	35.8 ± 1.42	32.4 ± 1.54	67.6 ± 1.05 a	13.0 ± 0.34
T2	37.2 ± 0.33	33.6 ± 1.15	64.2 ± 0.62 ab	12.6 ± 0.16
T3	35.1 ± 0.92	34.9 ± 1.56	62.4 ± 0.94 b	12.7 ± 0.18
T4	36.3 ± 1.43	36.5 ± 1.45	54.4 ± 1.41 c	12.3 ± 0.02

Note: ^†^ NS = not significant at *p* < 0.05 and * significant at *p* < 0.05; ^‡^ Values (mean of 3 replications) followed by the same letter, within the same column and factor, were not significantly different (*p* < 0.05), according to Tukey’s multiple range test.

**Table 3:** Profile of volatile compounds in almonds, retention index, and main odor and aroma descriptors.

Compound	Chemical Family	Code	RT (min)	Retention Index ^†^		Odor Descriptor
				Experimental	Literature ^‡^	
Ethanol	Alcohol	V1	2.235		489	Strong alcoholic ethereal medical [36]
2-Butanol	Alcohol	V2	2.363		608	Sweet apricot [36]
Hexane	Alkane	V3	2.861		600	Petroleum like [37]
3-Methyl furan	Furan	V4	2.986		646	
Acetic acid	Acid	V5	3.217		663	Pungent acidic cheesy vinegar [36]
3-Methyl-2-butenol	Alcohol	V6	4.864	761	770	Sweet fruity, green lavender [36]
1-Pentanol	Alcohol	V7	4.959	763	761	Pungent, fermented, bready, yeasty, winey, solvent [36]
2-Methyl-1-butanol	Alcohol	V8	5.048	765	748	Roasted, wine, onion, fruity, fusel, alcoholic, whiskey [36]
3-Methyl-1-butanol	Alcohol	V9	5.190	768	768	Fusel, alcoholic, cognac, fruity, banana, molasses [36]
2,3,3-Trimethyl pentane ^¥^	Alkane	V10	5.361	771	768	
2,2,5-Trimethyl hexane ^¥^	Alkane	V11	6.059	787	789	
2-Hexanol	Alcohol	V12	6.675	801	801	Chemical, winey, fruity, fatty, terpenic, cauliflower [36]
Hexanal	Aldehyde	V13	6.763	803	803	Fresh green fatty aldehydic grassy leafy fruity sweaty [36]
1-Hexanol	Alcohol	V14	9.795	870	869	Ethereal, fusel, oily, fruity, alcoholic, sweet, green [36]
Nonane	Alkane	V15	11.198	901	900	Gasoline [36]
2-Heptanol	Alcohol	V16	11.551	907	906	Fresh, lemongrass, herbal, sweet, floral, fruity, green [36]
Benzaldehyde	Aldehyde	V17	14.982	962	962	Almond, fruity, powdery, nutty, cherry, sweet, bitter [36]
2,2,4,6,6-Pentamethylheptane ^¥^	Alkane	V18	16.650	989	997	
Decane	Alkane	V19	17.461	1001	1000	
Limonene	Terpene	V20	19.416	1029	1029	Citrus, orange, sweet, fresh, peely [36]
Benzyl alcohol	Alcohol	V21	20.062	1038	1040	Sweet, floral, fruity, rose, balsamic nuances [36]
Undecane	Alkane	V22	24.615	1101	1100	
Nonanal	Aldehyde	V23	24.994	1107	1107	Waxy, aldehydic, citrus, green lemon peel, orange peel [36]
Dodecane	Alkane	V24	31.945	1201	1200	
Tridecane	Alkane	V25	39.090	1301	1300	
β-Damascone ^¥^	Ketone	V26	44.816	1385	1383	Fruity, floral, berry, plum, black currant, honey, rose, tobacco [36]

Note: ^¥^ = tentatively identified (identification only based on spectral database); ^†^ RT = retention time; ^‡^ = NIST (National Institute of Standards and Technology) [38].

**Table 4:** Volatile compounds (mg kg^−1^) found in raw almonds as affected by water stress. The quantification of these volatile compounds is based on the use of β-ionone as internal standard.

Code	Chemical	ANOVA ^†^	T1	T2	T3	T4
			(mg kg^−1^)			
V1	Ethanol	NS	0.11	0.21	0.17	0.16
V2	2-Butanol	NS	0.06	0.08	0.09	0.07
V3	Hexane ^¥^	NS	0.02	0.05	0.04	0.02
V4	3-Methyl furan	*	0.01 b	0.02 a	0.01 b	0.01 b
V5	Acetic acid	NS	0.03	0.05	0.04	0.02
V6	3-Methyl-2-butenol	NS	0.02	0.04	0.04	0.02
V7	1-Pentanol	NS	0.05	0.10	0.07	0.05
V8	2-Methyl-1-butanol	NS	0.05	0.08	0.05	0.04
V9	3-Methyl-1-butanol	NS	0.01	0.02	0.02	0.01
V10	2.3.3-Trimethyl pentane ^¥^	NS	0.01	0.02	0.03	0.02
V11	2.2.5-Trimethyl hexane ^¥^	NS	0.03	0.07	0.09	0.06
V12	2-Hexanol	NS	0.04	0.09	0.07	0.12
V13	Hexanal	NS	0.08	0.11	0.13	0.06
V14	1-Hexanol	***	0.31 ab	0.40 a	0.16 c	0.25 bc
V15	Nonane	NS	0.03	0.06	0.09	0.08
V16	2-Heptanol	NS	0.01	0.03	0.03	0.01
V17	Benzaldehyde	NS	0.03	0.06	0.04	0.04
V18	2,2,4,6,6-Pentamethyl heptane ^¥^	**	0.11 b	0.23 ab	0.46 a	0.50 a
V19	Decane	NS	0.23	0.48	0.39	0.42
V20	Limonene	*	0.04 ab	0.09 a	0.05 ab	0.03 b
V21	Benzyl alcohol	NS	0.02	0.05	0.02	0.03
V22	Undecane	NS	0.52	0.66	0.52	0.53
V23	Nonanal	NS	0.18	0.32	0.23	0.20
V24	Dodecane	NS	0.29	0.54	0.39	0.45
V25	Tridecane	***	0.16 c	0.45 ab	0.54 a	0.25 bc
V26	β-Damascone ^¥^	NS	0.06	0.08	0.06	0.06
	TOTAL	**	2.50 b	4.39 a	3.85 ab	3.52 ab

Note: ^†^ NS = not significant at *p* < 0.05; *. **. ***, significant at *p* < 0.05., 0.01., and 0.001, respectively; ^‡^ values (mean of 4 replications) followed by the same letter within the same row were not significantly different (*p* < 0.05), according to Tukey’s least significant difference test; ^¥^ = tentatively identified.

**Table 5:** Pearson’s correlation coefficients (*R*) among parameters.

	SI	Glucose	Malic Acid	Oleic Acid	Linoleic Acid	TPC	2-Butanol	Hexanal	Nonanal	TVC
Stress integral (SI)	**1.00**									
Glucose	−0.30	**1.00**								
Malic acid	0.21	−0.89	**1.00**							
Oleic acid (C18:1n9)	0.25	0.76	−0.89	**1.00**						
Linoleic acid (C18:2 n6)	0.57	0.50	−0.68	0.94 *	**1.00**					
Total phenolic Content (TPC)	0.72 **	−0.06	0.06	−0.08	0.18	**1.00**				
2-Butanol (V2)	0.57 *	−0.12	−0.20	0.19	0.18	0.29	**1.00**			
Hexanal (V13)	0.74	−0.01	−0.36	0.65 *	0.83 *	0.53	0.79	**1.00**		
Nonanal (V23)	0.75	0.32	−0.30	0.68 *	0.84 *	0.61	0.65	0.61	**1.00**	
Total volatile compounds (TVC)	0.75 ***	−0.02	0.23	0.02	0.37	0.76 ***	0.53 *	0.65 **	0.53 *	**1.00**

Note: *, **, ***, significant at *p* < 0.05, 0.01, and 0.001, respectively.
